# Structural basis of self-assembly in the lipid-binding domain of mycobacterial polar growth factor Wag31

**DOI:** 10.1107/S2052252520006053

**Published:** 2020-06-30

**Authors:** Komal Choukate, Barnali Chaudhuri

**Affiliations:** a GN Ramachandran Protein Center, CSIR Institute of Microbial Technology, Chandigarh, 160036, India; b Academy of Scientific and Innovative Research (AcSIR), Anusandhan Bhawan, 2 Rafi Marg, New Delhi, 110001, India

**Keywords:** mycobacterial polar growth, dimer assembly, mycobacterium tuberculosis, coiled coil, lipids, filaments

## Abstract

Wag31, or DivIVA, is an essential protein and a drug target in the human pathogen *Mycobacterium tuberculosis* that self-assembles at the negatively curved membrane surface to form a higher-order structural scaffold, maintains rod-shaped cellular morphology and localizes key cell-wall synthesizing enzymes at the pole for exclusive polar growth. The crystal structure of the N-terminal lipid-binding domain of mycobacterial Wag31 was determined at 2.3 Å resolution. The structure revealed a highly polar surface lined with several conserved charged residues that suggest probable sites for interactions with membrane lipids. Crystal-packing analysis revealed a previously unseen ‘dimer-of-dimers’ assembly state of N-terminal Wag31, which is formed by antiparallel stacking of two coiled-coil dimers. Size-exclusion column-chromatography-coupled small-angle solution X-ray scattering data revealed a tetrameric form as a major assembly state of N-terminal Wag31 in solution, further supporting the crystal structure. The results suggest that, in addition to lipid binding, the N-terminal Wag31 can participate in self-assembly to form filamentous structures. Plausible models of linear self-assembly and branching of Wag31 filaments consistent with available data are suggested.

## Abstract 2

The crystal structure of the N-terminal membrane anchoring domain of mycobacterial DivIVA/Wag31 reveals a filament-compatible ‘dimer-of-dimers’ assembly state. The results suggest that, in addition to lipid binding, the N-terminal of Wag31 can participate in self-assembly to form filamentous structures.

## 1. Introduction   

The sensing of membrane curvature plays a critical role in diverse physiological processes such as the maintenance of cellular morphology, polar or hyphal growth in bacteria and endocytosis in eukaryotes (Cannon *et al.*, 2017 ▸). Prominent examples of positive curvature-sensing proteins include septins and BAR (Bin/amphiphysin/Rvs) domain proteins in eukaryotes, and SpoVM in sporulating bacteria (Cannon *et al.*, 2017 ▸). In Gram-positive bacteria, DivIVA recognizes negative concave membrane curvature, and self-assembles at the cytoplasmic side of the pole and curved region of the cell-division septum to form a structural scaffold (Edwards & Errington, 1997 ▸; Letek *et al.*, 2008 ▸; Lenarcic *et al.*, 2009 ▸; Ramamurthi & Losick, 2009 ▸; Kaval & Halbedel, 2012 ▸; Bach *et al.*, 2014 ▸). DivIVA, which is a filamentous coiled-coil protein, aids in the localization of various target proteins at the pole for cell-wall growth and a variety of biological functions (Rudner & Losick, 2010 ▸; Laloux & Jacobs-Wagner, 2014 ▸; Halbedel & Lewis, 2019 ▸; Hammond *et al.*, 2019 ▸).

DivIVA, also known as Wag31 and antigen 84, is essential in mycobacteria, and is required for its exclusive polar growth, morphology maintenance and other functions (Nguyen *et al.*, 2007 ▸; Kang *et al.*, 2008 ▸; Mukherjee *et al.*, 2009 ▸; Jani *et al.*, 2010 ▸; Plocinski *et al.*, 2012 ▸; Ginda *et al.*, 2013 ▸; Plocinska *et al.*, 2014 ▸; Melzer *et al.*, 2018 ▸). Furthermore, Wag31 is targeted by bactericidal amino-pyrimidine sulfonamides, though the mechanism of antibacterial action is not known (Boshoff, 2017 ▸; Singh *et al.*, 2017 ▸). Wag31 plays a critical role in regulating peptidoglycan biosynthesis and localizing many cell-wall synthesizing enzymes at the pole to support polar growth (Kang *et al.*, 2008 ▸; Jani *et al.*, 2010 ▸; Meniche *et al.*, 2014 ▸; Xu *et al.*, 2014 ▸). Wag31 is phospho­rylated at a single site (Thr73) that enables it to be better at localization at the pole and at regulation of peptidoglycan biosynthesis (Kang *et al.*, 2008 ▸; Jani *et al.*, 2010 ▸). A small membrane-bound protein CwsA may assist Wag31 localization at the mycobacterial pole (Plocinski *et al.*, 2012 ▸). While depletion of Wag31 leads to a ‘rod to spherical cell’ transition (Nguyen *et al.*, 2007 ▸; Kang *et al.*, 2008 ▸; Meniche *et al.*, 2014 ▸), Wag31 is shown to contribute to restoration of rod shape in spherical cells (Melzer *et al.*, 2018 ▸).

Oligomerization appears to be a recurring theme in the sensing of mesoscale (∼100 nm to 1 µm) curvature by nanoscale protein subunits in several cases (Antonny, 2011 ▸; Cannon *et al.*, 2017 ▸). A ‘molecular bridging of the curvature’ by oligomers of DivIVA was suggested as a mechanism of DivIVA binding to a concave membrane (Lenarcic *et al.*, 2009 ▸). However, how DivIVA oligomerizes is sparsely understood. Structural data on DivIVA is so far limited to only one study (Oliva *et al.*, 2010 ▸). In this study, a model of a 30 nm long tetrameric form of DivIVA from *Bacillus subtilis* (bsDivIVA) was proposed based on the crystal structures of individual N-terminal and C-terminal coiled-coil domains of DivIVA (Oliva *et al.*, 2010 ▸). Conserved phenylalanine and arginine residue pairs at both ends of this bent elongated tetrameric structure occupy the proposed sites of polar membrane tethering (Oliva *et al.*, 2010 ▸). In addition, super-resolution microscopy revealed double-ring structures formed by self-assembled bsDivIVA near the division septum (Eswaramoorthy *et al.*, 2011 ▸). Electron micrographs showing oligomerization of bsDivIVA mutants to form ∼22 nm long ‘doggy-bone’ shaped structures, and longer strings and networks *in vitro* were also reported (Stahlberg *et al.*, 2004 ▸). Recently, it was shown that the mycobacterial Wag31 (tbWag31) forms several micrometre long primarily linear polymers *in vitro* with occasional bending or branching (Choukate *et al.*, 2019 ▸). The structural basis of these oligomeric formations remains elusive.

Here, we report the crystal structure of the N-terminal lipid-binding domain of tbWag31 (N-Wag31) at 2.3 Å resolution. Crystal-packing analysis reveals a novel tetrameric form of N-Wag31 composed of two dimeric coiled-coil domains stacked in antiparallel fashion, which is compatible with linear filament formation. Accompanying size-exclusion columnchromatography-coupled small-angle X-ray scattering (SEC-SAXS) data further support a tetrameric form of N-Wag31 in solution. Our data suggest that, in addition to membrane lipid binding, the lipid-binding domain of Wag31 can participate in self-assembly. Based on the available data, we proposed possible models of Wag31 self-assembly for linear, and branched, filament formation.

## 2. Materials and methods   

### 2.1. Recombinant protein expression   

The N-terminal domain of Wag31 (residues 2–60 of tbWag31 with an N-terminal tag containing residues ‘MAHHHHHHENLFYQG’, the codon-optimized gene synthesized and sub-cloned in a pET15b vector by Genescript) construct was expressed in *Escherichia coli* BL21 (DE3) cells. A primary culture of volume 20 ml was started at 37°C in LB broth containing 100 mg ml^−1^ ampicillin and incubated overnight. This primary culture was transferred and grown in 2 l of LB broth at 37°C until an OD_600_ of 0.6 was reached. Next, recombinant protein expression was induced by adding 1 m*M* IPTG, and cells were incubated at 25°C for 16 h. The cells were harvested and stored at −80°C for further use.

### 2.2. Protein purification   

The frozen cell pellets were resuspended in lysis buffer containing 20 m*M* Tris-HCl pH 7.0 and 150 m*M* NaCl with 0.2% Triton-X 100 and then lysed by sonication. The cell lysate was loaded onto a nickel IMAC Sepharose fast-flow affinity column (GE healthcare) and bound protein was eluted with a buffer containing 50 m*M* Tris–HCl at pH 7.0, 150 m*M* NaCl and 500 m*M* imidazole. The fractions of interest were pooled, concentrated and further purified by size-exclusion chromatography using a HiLoad 16/60 Superdex 200 pg column (GE healthcare) in the following buffer: 50 m*M* Tris–HCl at pH 7.0 and 150 m*M* NaCl. Freshly purified N-Wag31 protein was concentrated to 10 mg ml^−1^ for crystallization experiments.

### 2.3. Circular dichroism spectropolarimetry   

For secondary structure estimation, CD spectra of purified N-Wag31 at ∼1 mg ml^−1^ concentration was measured at 25°C using an in-house Jasco J815 spectropolarimeter. Three CD spectra scans were averaged and plotted as molar-residue ellipticity versus wavelength.

### 2.4. Crystallization and data collection   

Crystals of N-Wag31 were grown by both hanging- and sitting-drop vapour-diffusion methods at 19°C in 0.2 *M* magnesium chloride, 0.1 *M* sodium cacodylate at pH 6.5 and 50%(*v*/*v*) polyethyl­ene glycol 200. These crystals were very small and recalcitrant to growth. Crystals were flash frozen in liquid nitro­gen without any additional cryo-protectant and transported to the European Synchrotron Radiation Facility (ESRF) in France. X-ray diffraction data from an N-Wag31 crystal were collected at the microfocus beamline ID30A-3 (MASSIF-3) at ESRF, which is suitable for such small crystals. Only one useful dataset was obtained after testing 37 crystals. Data collection was carried out at 100 K, using an EIGER detector (DECTRIS Ltd). Data indexing, processing, merging and scaling were performed using *XDS* (Kabsch, 2010 ▸) and the *CCP*4 suite of software (Winn *et al.*, 2011 ▸).

### 2.5. Crystal structure determination, model building and refinement   

The crystal structure of N-Wag31 was determined using the molecular replacement method (*MOLREP*, Vagin & Teplyakov, 1997 ▸). The crystal structure of its homolog N-bsDivIVA (PDB code 2wuj; Oliva *et al.*, 2010 ▸) was used as a search model in a dimeric form, after converting it to a poly-alanine model. Following rigid-body minimization of the best solution, positional refinements were performed using *REFMAC* (Murshudov *et al.*, 1997 ▸) with non-crystallographic symmetry (NCS) restraints. An *R*_free_ set was used to monitor the progress of refinement and individual isotropic *B* factors were refined. Model rebuilding was performed using *Coot* (Emsley *et al.*, 2010 ▸). *SIGMAA*-weighted difference maps and composite OMIT maps were used for model rebuilding. In the latter stages of refinement, simulated-annealing runs were performed in *Phenix* (Adams *et al.*, 2010 ▸). NCS restraints were also released during the final rounds of the refinement. In addition to two protein chains and water molecules, the asymmetric unit contains half of a tri­ethyl­ene glycol (PGE) related to itself by a twofold crystallographic symmetry operation. *MOLPROBITY* was used for structure validation (Chen *et al.*, 2010 ▸).

**Figure 1:**
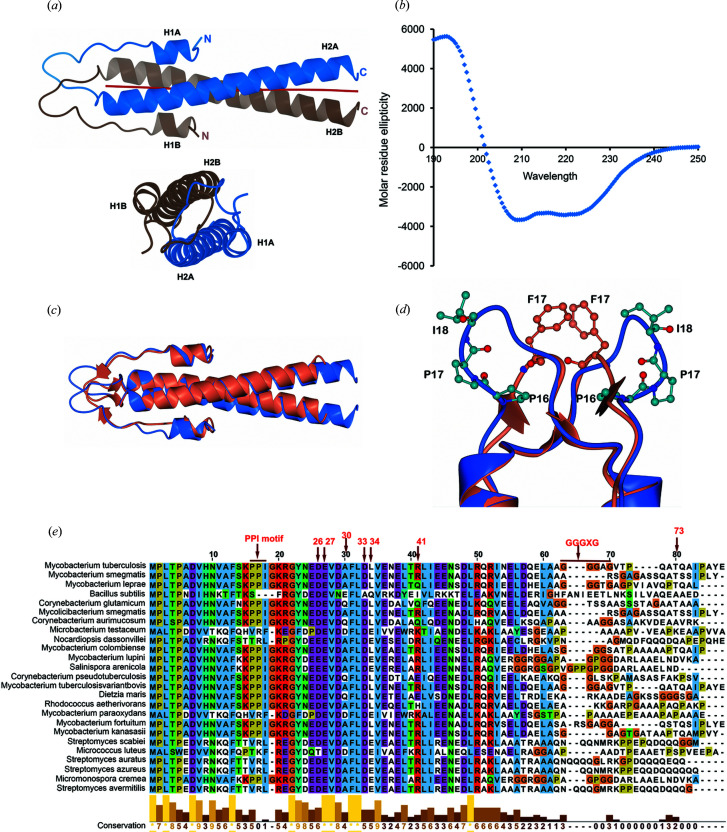
Crystal structure of N-Wag31. (*a*) Two nearly orthogonal views of the N-Wag31 (residues 2–60) dimer as blue and pale brown ribbons are shown. The calculated local coiled-coil axes are shown as red jointed lines. (*b*) A CD profile (molar-residue ellipticity versus wavelength) of N-Wag31. (*c*) Superimposed structures of N-Wag31 (blue) and N-bsDivIVA (coral) are shown. (*d*) The intertwined loop region in N-Wag31 (blue) containing the P_16_P_17_I_18_ sequence (balls and sticks, with carbon atoms in light blue, oxygen in red and nitro­gen in blue) and the equivalent region in N-bsDivIVA (coral) containing the stacked Phe17 residues (balls and sticks, with carbon atoms in coral, oxygen in red and nitro­gen in blue), are shown. (*e*) Multiple sequence alignment of the N-terminal region of tbWag31 (numbering for tbWag31 in red) and homologs, with the degree of conservation, is shown (prepared in *Jalview*; Waterhouse *et al.*, 2009 ▸).

N-Wag31 contains two chains, A and B, in the asymmetric unit [Figs. 1 ▸(*a*) and S1 in the Supporting information]. In addition to residues 2–60, chain A contains a large, contiguous segment of the poly-histidine tag region (residues −9 to 1). Chain B contains residues 2–60. An isolated tripeptide interacting with a neighbouring molecule, tentatively containing ‘HHE’ sequence, was assigned to chain B. However, as this tripeptide segment is far from the rest of the B chain, an out of register error may not be ruled out. Electron density at and around the loop region containing residues 17–20 was rather poor, especially in chain A. The main-chain conformation for this loop region was traced and built in the electron-density map for chain B and rebuilt in the same way for chain A. Side-chain densities were poor for several residues around this loop region and at the C-terminal end of N-Wag31, which is reflected in higher than average temperature factors for these side chains. All these side chains were modelled using suitable rotamers from the *Coot* rotamer library (Lovell *et al.*, 2000 ▸). Loss of side-chain density could be a result of radiation damage caused by X-rays at the microfocus beamline or flexibility. Data-collection and refinement statistics are summarized in Table 1 ▸. Figures were prepared using *PyMOL* (*The PyMOL Molecular Graphics System*, Schrodinger, LLC) and *CCP4MG* from the *CCP*4 suite (Winn *et al.*, 2011 ▸).

### 2.6. Size-exclusion chromatography coupled with SAXS experiment   

The SEC-SAXS experiments were performed at the BM29 beamline at ESRF using a Shimadzu HPLC system. The purified N-Wag31 (4.4 mg ml^−1^) in a buffer containing 20 m*M* Tris pH 7.5, 150 m*M* NaCl and 10% glycerol was used for the SAXS experiments. The protein was snap frozen under liquid nitro­gen and transported to the synchrotron site, where it was thawed on ice prior to the experiments. Furthermore, 30 µl of protein sample was injected into an Agilent Bio-SEC-3 column. SEC-SAXS data were collected at ∼1 Å X-ray wavelength, with 1 s frame^−1^ exposure and 2.8 m detector distance, using a Pilatus detector (Dectris Ltd). SEC-SAXS data analysis was performed using the *ATSAS* suite of software (Franke *et al.*, 2017 ▸).

### 2.7. Data availability   

Protein structure coordinates and structure factors have been deposited in the PDB with accession code 6lfa. SAXS data for peak II are available from SASBDB with accession code SASDHH4.

## 3. Results   

### 3.1. Crystal structure of the N-terminal domain of Wag31   

Mycobacterial Wag31 (P9WMU1, Rv2145c) is a 260-residue long filament-forming protein containing two domains: an N-terminal lipid- or membrane-binding domain and a C-terminal domain that participates in polar protein localization. The crystal structure of the N-terminal domain of tbWag31, or N-Wag31, shows a parallel coiled-coil dimer composed of two chains, A and B, containing residues 2–60 and additional residues from the poly-histidine tag regions [Fig. 1 ▸(*a*), see Fig. S1 and the experimental methods section[Sec sec2]]. The two chains of N-Wag31 are similar to each other, with a root-mean-square deviation (RMSD) of 0.65 Å for 59 Cα atoms (calculated using *CLICK*; Nguyen *et al.*, 2011 ▸). The N-terminal segment of N-Wag31 contains a short helical turn (H1 helix) and loop followed by a sharp turn joining the coiled-coil helix (H2 helix). The loop region is intertwined in the N-Wag31 dimer, as observed for the N-terminal domains of its structural homologs bsDivIVA (N-bsDivIVA) and GpsB (Oliva *et al.*, 2010 ▸; Halbedel & Lewis, 2019 ▸; Cleverley *et al.*, 2019 ▸). CD data obtained from N-Wag31 supported an α-helix rich N-Wag31 structure in solution, with an estimated helical content of ∼60% [Fig. 1 ▸(*b*), calculated using *K2D*; Andrade *et al.*, 1993 ▸]. The average coiled-coil pitch is ∼171 Å for residues 26–60 of N-Wag31 (calculated using *TWISTER*; Strelkov & Burkhard, 2002 ▸).

THe N-Wag31 structure is similar to the structure of its homolog N-bsDivIVA, with which it shares ∼42% sequence identity, with a 1.6 Å RMSD for 56 Cα atoms in a chain [Fig. 1 ▸(*c*), computed using *Dali* (Holm, 2019 ▸)]. However, the two structures differ significantly in the intertwined loop region that houses the lipid-binding site [Figs. 1 ▸(*c*) and 1 ▸(*d*)]. In the N-bsDivIVA structure, two spatially adjacent phenylalanine residues (Phe17) from the intertwined loops of two subunits in the coiled-coil dimer were shown to participate in lipid binding [Fig. 1 ▸(*d*); Oliva *et al.*, 2010 ▸; Halbedel & Lewis, 2019 ▸]. The putative lipid-binding region in N-Wag31 houses a conserved ‘P_16_P_17_I_18_’ motif instead of Phe17 [Figs. 1 ▸(*d*) and 1 ▸(*e*)]. Exposed non-polar Ile18 at the tip of the intertwined loop is a candidate for direct interaction with membrane lipid. However, distances between the Cα atoms of the two Ile18 residues in N-Wag31 are ∼15 Å. In comparison, the Cα atoms of lipid-binding Phe17 residues in N-bsDivIVA are separated by ∼5 Å, with their side-chain aromatic rings stacked [Fig. 1 ▸(*d*)]. A conformational change may possibly bring the Ile18 hydro­phobic side chains together in N-Wag31 to form a lipid-interacting patch. On the other hand, the presence of two proline residues before Ile18 makes this region structurally rigid. Thus, the putative lipid-binding region is quite different in N-Wag31 than in N-bsDivIVA.

### 3.2. Crystal-packing analysis suggests a ‘dimer-of-dimers’ organization of N-Wag31   

**Figure 2:**
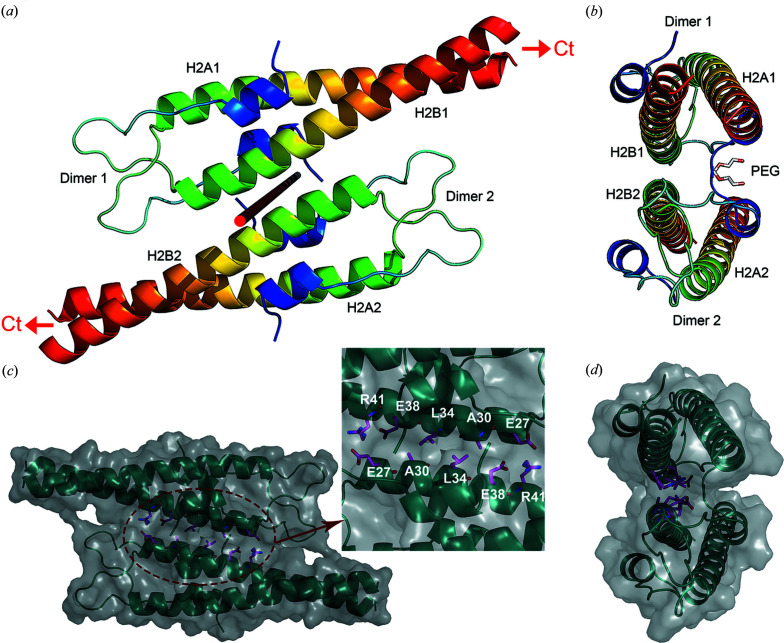
Oligomeric organization of N-Wag31. (*a*), (*b*) Two views of the ‘dimer-of-dimers’ form of N-Wag31 related by crystallographic twofold symmetry are shown as ribbons (Jones rainbow, blue to red). The coiled-coil forming H2 helices in both A and B chains in both dimers are labelled as follows: H2A1 and H2B1 in A and B chains of dimer 1, and H2A2 and H2B2 in A and B chains of dimer 2. The directions to the C-terminal domains (Ct) are marked with red arrows in (*a*). The twofold axis of rotation is shown as a red rod in (*a*). A PGE molecule bound to N-Wag31 is shown as sticks in (*b*). (*c*), (*d*) Two views of the ‘dimer-of-dimers’ of N-Wag31 are shown as ribbons (cyan) within the semi-transparent molecular surface (grey) of the tetramer. Side chains of the interface-forming residues are shown as balls and sticks (carbon in magenta, oxygen in red and nitro­gen in blue). A zoomed view of the side-chain residues (Arg41, Glu27, Leu34, Ala30 and Glu38) at the interface is shown in the inset.

Coiled coils can assemble in a number of ways to form higher-order assemblies (Moutevelis & Woolfson, 2009 ▸). In order to learn about assembly of N-Wag31, we performed crystal-packing analysis. It appears that the dimeric coiled-coil domain of N-Wag31 combines with an adjacent crystallographic twofold-symmetry-related dimer to form a tetramer [Figs. 2 ▸(*a*) and 2 ▸(*b*)]. Isologous interface in N-Wag31 was assigned using the *PDBePISA* server (Krissinel & Henrick, 2007 ▸). The tetramer buries ∼396.0 Å^2^ of water-accessible surface area upon assembly, with the coiled-coil forming H2 helices of the two B chains with complimentary surface shapes stacking against each other [Figs. 2 ▸(*c*) and 2 ▸(*d*)]. The two stacked H2 helices are in near-antiparallel orientation, with an angle of ∼176° between the two local helix axes (computed using residues 26–41).

The size of the buried surface area is a critical quantity that helps in distinguishing between crystal contacts and biological interfaces (∼370–4750 Å^2^ for a homodimer; Henrick & Thornton, 1998 ▸; Krissinel & Henrick, 2007 ▸). The buried surface area at the crystallographic twofold-related interface in N-Wag31 is small (∼396.0 Å^2^). This crystallographic interface is lined by residues that are conserved in many actinobacteria, such as Asp26, Glu27, Ala30, Leu34 and Arg41 [Fig. 2 ▸(*c*)]. The center of this interface is formed by buried non-polar Leu34 and Ala30. Two salt bridges are formed between the side chains of Glu27 and Arg41 residues at this interface on both sides of the central non-polar region [Fig. 2 ▸(*c*)]. A second buried surface area (<300 Å^2^) was detected at the C-terminal end of N-Wag31, which is probably formed because of crystal packing. In addition, a PEG molecule was found buried at one side of this assembly interface, covering a rather small, ∼80 Å^2^, part of the accessible surface area of the N-terminal H1 helix [Fig. 2 ▸(*b*)]. Note that formation of a tetrameric form of N-Wag31 in the absence of PEG is supported by solution SAXS data, which are described in the next section.

### 3.3. Solution scattering supports a ‘dimer-of-dimers’ organization of N-Wag31   

**Figure 3:**
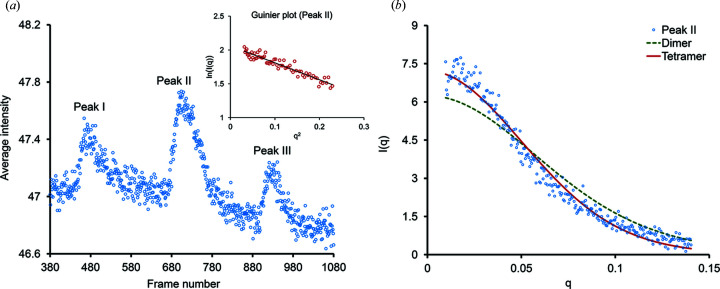
SEC-SAXS data analysis. (*a*) A SAXS elution profile (average intensity versus frame number obtained from *CHROMIXS*; Franke *et al.*, 2017 ▸) of N-Wag31. The Guinier plot [ln*I*(*q*) versus *q* ^2^, *I* is intensity and *q* is momentum transfer in nm^−1^] for peak II is shown in the inset. (*b*) A scattering profile of peak II data (*I* versus *q* in Å^−1^). Computed scattering profiles of dimeric and tetrameric N-Wag31 (calculated using *CRYSOL* with suggested parameters; Franke *et al.*, 2017 ▸) are shown fitted to the experimental data.

In order to determine the solution assembly states of N-Wag31, we performed a SEC-SAXS experiment. The size-exclusion column elution profile of N-Wag31 revealed the presence of multiple assembly states eluted as separate peaks, numbered from left to right as peaks I, II and III, respectively [Figs. 3 ▸(*a*) and S2(*a*)]. The early eluting peak I suggests the presence of large aggregates [Figs. 3 ▸(*a*) and S2(*b*)]. The averaged SAXS data obtained from peak III, which we expect to be a dimer, were too weak for analysis. SAXS data analysis suggests that the peak II region contains a tetrameric form of N-Wag31 (∼8.6 kDa monomer^−1^), with a predicted mass of ∼34.6 kDa based on the Bayesian inference method (Hajizadeh *et al.*, 2018 ▸). The central peak II appears to be the major assembly state in solution [Fig. S2(*a*)]. Residues −9 to 1 of chain A of N-Wag31 containing a near-complete poly-histidine tag were used to build an equivalent region of chain B of the dimer, and the tetramer, using NCS operations, for all SAXS calculations. However, this tag region might be floppy in solution. The radius of gyration (*R*_g_) obtained from the Guinier analysis of averaged SAXS data from peak II was 27.2 Å [*q* *R*_g_ ≤ 1.3, Fig. 3 ▸(*a*), where *q* refers to momentum transfer in nm^−1^], while *R*_g_ calculated from dimeric/tetrameric N-Wag31 coordinates was ∼20.9 and 24.2 Å, respectively. χ^2^ values for fits between the theoretical scattering intensities derived from the tetrameric and dimeric N-Wag31 models with experimental averaged scattering intensity from peak II were 1.45 and 4.0, respectively, further supporting the tetrameric structure [Fig. 3 ▸(*b*)]. As the averaged SAXS data were quite noisy in the high-angle region and inadequate for further analysis, shape reconstructions were not performed. To summarize, solution SEC-SAXS data support a tetrameric assembly of N-Wag31, which is consistent with the crystal structure.

### 3.4. Surface-charge distribution suggests putative lipid-binding sites in N-Wag31   

**Figure 4:**
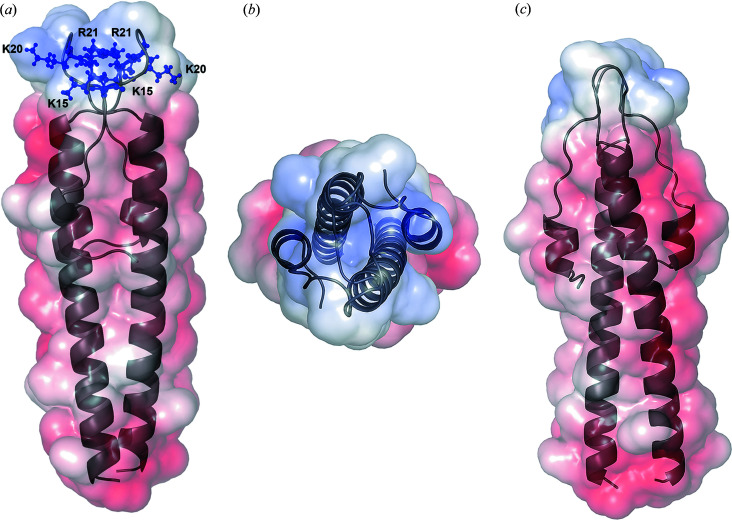
Surface-charge distribution in N-Wag31. (*a*)–(*c*) Different views of the dimeric N-Wag31 (grey) with electrostatic potential mapped into the semi-transparent water-accessible surface (a water radius of 1.4 Å) are shown. Conserved positively charged residues (Lys15, Lys20, Arg21) at the lipid-interacting intertwined loop region of N-Wag31 are shown as blue balls and sticks in (*a*). Electrostatic potential was computed using *APBS* with *AMBER* charges and default parameters (Baker *et al.*, 2001 ▸).

Mapping the Poisson–Boltzmann electrostatic potential into the solvent-accessible surface revealed that the surface of the lipid-binding N-Wag31 dimer is highly polar [Figs. 4 ▸(*a*)–4 ▸(*c*)]. The N-Wag31 sequence contains 14 negatively charged and six positively charged residues. The surface of the homologous N-bsDivIVA, with several charged residues, is similarly polar (Oliva *et al.*, 2010 ▸). This is not surprising as charged amino acids are more common in coiled coils than other proteins (Surkont & Pereira-Leal, 2015 ▸). The electrostatic potential calculation shows a positively charged patch at the intertwined loop region of N-Wag31, which is lined with conserved arginine and lysine residues [Figs. 4 ▸(*a*) and 4 ▸(*b*)]. This region probably defines the conserved positively charged membrane-associating surface in the Wag31/DivIVA family of proteins and GpsB that interact with membrane phospho­lipids (Oliva *et al.*, 2010 ▸; Killian & Heijne, 2000 ▸; Halbedel & Lewis, 2019 ▸).

In addition to the positively charged intertwined loop region, the tetrameric form of N-Wag31 contains negatively charged patches on its water-accessible surface [Figs. 4 ▸(*a*)–4 ▸(*c*)]. Negatively charged protein residues can interact with ethano­lamine lipids in the membrane (Jurásek *et al.*, 2019 ▸). Ethano­lamine is present in the mycobacterial membrane (Chiaradia *et al.*, 2017 ▸) and can potentially interact with conserved negatively charged surface patches in the tetrameric N-Wag31.

### 3.5. A suggested model for Wag31 filament formation   

**Figure 5:**
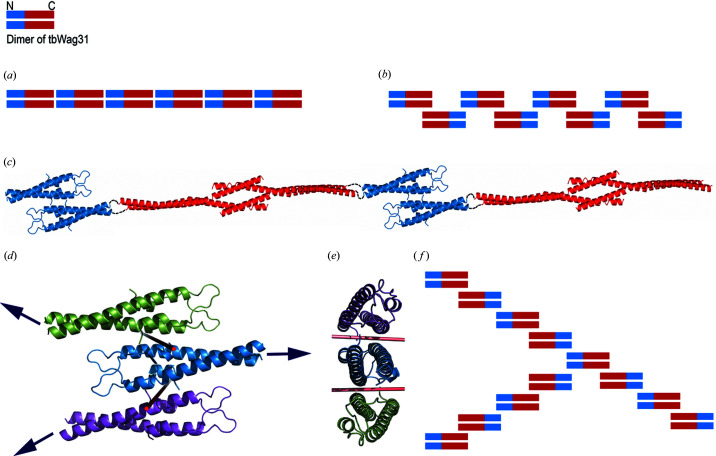
A proposed model of Wag31 filament assembly. (*a*), (*b*) Two theoretically alternative possible modes of self-assembly of Wag31, using (*a*) pure translational repeat operations and (*b*) twofold-symmetry-related repeat operations, are shown. (*c*) Ribbon diagrams of the tetramers of N-terminal N-Wag31 (blue, this work) and the C-terminal domain of bsDivIVA (red, Oliva *et al.*, 2010 ▸) are shown, joined by linker regions as dashed lines, as a theoretical filament. The linker region is ∼20 residues long in bsDivIVA, and ∼100 residues long in tbWag31. (*d*), (*e*) Two views of a theoretical hexamer composed of three dimers of N-Wag31 (ribbons in green, blue and magenta). The twofold axes of rotation are shown as red rods. (*f*) A theoretically possible mode of 3-way branching in tbWag31 filament.

Linear filaments, with sporadic branching, were recently reported for full-length tbWag31 (Choukate *et al.*, 2019 ▸). The average diameters of these full-length tbWag31 filaments (1.4–2.1 nm, Choukate *et al.*, 2019 ▸) are roughly similar to the diameter of a coiled coil, which is ∼2 nm. The N-Wag31 forms a ‘dimer-of-dimers’ with the C-terminal regions extending in opposite directions, which is compatible with such linear filament formation [Fig. 2 ▸(*a*)]. A protein subunit can be arranged to form a filament in a few ways [Figs. 5 ▸(*a*) and 5 ▸(*b*)]. It could polymerize by a pure translational repeat along a certain direction or it could polymerize by a combination of rotation and translation along a helical axis. Alternatively, a protein subunit can form a filament by utilizing twofold symmetry operations. For a protein with an N-terminal domain N and a C-terminal domain C, two such theoretical arrangements would be —NC—NC—NC—NC—NC— or —NC—CN—NC—CN—NC— [Figs. 5 ▸(*a*) and 5 ▸(*b*)]. In the latter case, N—N and C—C inter-subunit interfaces related by twofold symmetry would be formed [Fig. 5 ▸(*b*)]. Wag31 appears to form a linear filament using the second option involving twofold symmetry operations [Fig. 5 ▸(*c*)].

The structure of the C-terminal domain of tbWag31, which is predicted to be a coiled coil (Choukate *et al.*, 2019 ▸), is currently not available. A low-resolution model of the C-terminal domain of bsDivIVA was shown to form a ‘dimer-of-dimers’, with a central 4-helix bundle region [Fig. 5 ▸(*c*); Oliva *et al.*, 2010 ▸]. The sequence identity between this truncated C-terminal domain of bsDivIVA and that of the C-terminal region of tbWag31 (residues 165–234) is about 26%, suggesting that the C-terminal Wag31 may assume a bsDivIVA-like ‘dimer-of-dimers’ structure. A combination of the two such N-terminal and C-terminal ‘dimer-of-dimers’ can be used to build a linear filament of Wag31 [Fig. 5 ▸(*c*)].

Furthermore, the location of the twofold-symmetry-related interface in the N-Wag31 suggests a natural way for lateral or side-way association of dimeric N-Wag31 units to form higher-order oligomers, such as a hexamer [Figs. 5 ▸(*d*)–5 ▸(*f*)]. Such a theoretical hexameric association of N-Wag31 can lead to branching or bifurcations in a Wag31 filament (amongst other possibilities), and can be exploited for protein-based scaffold design [Figs. 5 ▸(*d*) and 5 ▸(*e*)].

## 4. Conclusions   

The crystal structure of N-Wag31 reported here reveals a tetrameric form of N-Wag31, which is further supported by solution SAXS data. In contrast, homologous N-bsDivIVA is reportedly a dimer in solution (Oliva *et al.*, 2010 ▸). A tetrameric form of bsDivIVA was built by combining the crystal structures of C-terminal ‘dimer-of-dimers’ and two terminally located dimeric N-terminal domains (Oliva *et al.*, 2010 ▸). However, how such a tetramer can be further assembled to build higher-order structures was not obvious. Based on electron micrographs of filaments formed by bsDivIVA mutants, an assembly pathway of ‘doggy-bone’ shaped basic building blocks were suggested (Stahlberg *et al.*, 2004 ▸). However, atomic details of such assembly remained elusive. The tetrameric form of N-Wag31 reported here elucidates the structural basis of such assembly formation involving the N-terminal lipid-binding domain that can lead to higher-order structures. Furthermore, considering that tbWag31 is a high-confidence drug target (Singh *et al.*, 2017 ▸; Boshoff, 2017 ▸), the structural data presented here can be exploited for designing inhibitors.

How N-Wag31 orients itself at the polar membrane surface for curvature sensing is currently not known. Molecular-dynamics simulation studies suggested that lipid composition can influence the orientation of DivIVA at the membrane (Jurásek *et al.*, 2019 ▸). Ethano­lamine lipids may interact with negatively charged surface patches causing DivIVA to realign from perpendicular to parallel orientation at the membrane surface (Jurásek *et al.*, 2019 ▸). Likewise, N-Wag31 orientation may be determined by interactions involving both the positively charged intertwined loop region and negatively charged surface patches [Figs. 4 ▸(*a*)–4 ▸(*c*)] with the polar membrane lipids.

The crystal structure of N-Wag31 that comprises residues 2–60 of tbWag31 raises questions about the possible structural role of phospho­rylated Thr73 residue. The Thr73 residue is in a putative intrinsically unstructured region adjacent to the N-terminal domain of Wag31 (Choukate *et al.*, 2019 ▸) and proximal to several glycine residues, such as G_63_G_64_G_65_X_66_G_67_ [Fig. 1 ▸(*e*)]. Small and flexibility-imparting glycine residues are typically rare in coiled coils (Surkont & Pereira-Leal, 2015 ▸). Within the classical heptad repeat ‘*abcdefg*’ in a coiled coil, the ‘*a*’ and ‘*d*’ regions are buried inside where ‘*g*’ and ‘*e*’ regions make ionic interactions between the helices to stabilize the coiled-coil assembly. If the coiled-coil heptad assignment (made by *TWISTER*; Strelkov & Burkhard, 2002 ▸) is theoretically extended beyond the last residue (Leu60, assigned ‘*a*’ position) observed in the crystal structure, the 73rd residue will be in the ‘*g*’ position assuming coiled-coil continuity, and could be critical for providing a stabilizing salt link for coiled-coil continuation in the phospho­rylated form of tbWag31 in an otherwise flexible region.

## Supplementary Material

Supporting figures. DOI: 10.1107/S2052252520006053/lz5036sup1.pdf

PDB reference: Structure of the N-terminal domain of Wag31, 6lfa

SASBDB reference: N-terminal domain of Wag31 from *M. tuberculosis*, SASDHH4

## Figures and Tables

**Table 1:** Crystal-diffraction and model-refinement statistics (generated using the Table 1 option of *Phenix*; Adams *et al.*, 2010 ▸) Statistics for the highest-resolution shell are shown in parentheses.

Diffraction-data and refinement statistics	N-Wag31
Experimental station	ID30A-3, ESRF
Wavelength (Å)	0.97
Resolution range (Å)	30.1–2.3 (2.4–2.3)
Space group	*C*121
Unit-cell parameters (*a*, *b*, *c* in Å and α, β, γ in °)	44.9, 53.8, 61.2, 90, 100.8, 90
Total reflections	77318 (5929)
Unique reflections	6374 (592)
Multiplicity	12.1 (9.9)
Completeness (%)	98.6 (92.2)
〈*I*/σ(*I*)〉	7.45 (2.2)
Wilson *B* factor (Å^2^)	21.75
*R*_merge_	0.235 (1.054)
*R*_meas_	0.245 (1.112)
*R*_pim_	0.070 (0.345)
CC_1/2_	0.99 (0.76)
CC^*^	0.99 (0.93)
Reflections used in refinement	6363 (592)
Reflections used for *R*_free_	353 (38)
*R*_work_	0.175 (0.180)
*R*_free_	0.225 (0.245)
Number of non-H atoms	1137
Macromolecules	1091
PGE	10
Water	36
Protein residues	132
RMS (bonds, Å)	0.010
RMS (angles, °)	1.05
Ramachandran favoured (%)	98.4
Ramachandran allowed (%)	1.6
Ramachandran outliers (%)	0.00
Rotamer outliers (%)	0.8
Average *B* factor (Å^2^)	32.6
Macromolecules	32.6
PGE	35.5
Water	31.3
