# Supplementary material for: Structural basis of self-assembly in the lipid-binding domain of mycobacterial polar growth factor Wag31
Source: IUCrJ. 2020 Jun 30;7(Pt 4):767–76. doi: 10.1107/S2052252520006053 (PMC7340271; doi:10.1107/S2052252520006053)
Supplement: Supplementary file 1 [file m-07-00767-sup1.pdf]

IUCrJ

Volume 7 (2020)

Supporting information for article:

Structural basis of self-assembling in the lipid-binding domain of mycobacterial polar growth factor Wag31

Komal Choukate and Barnali Chaudhuri

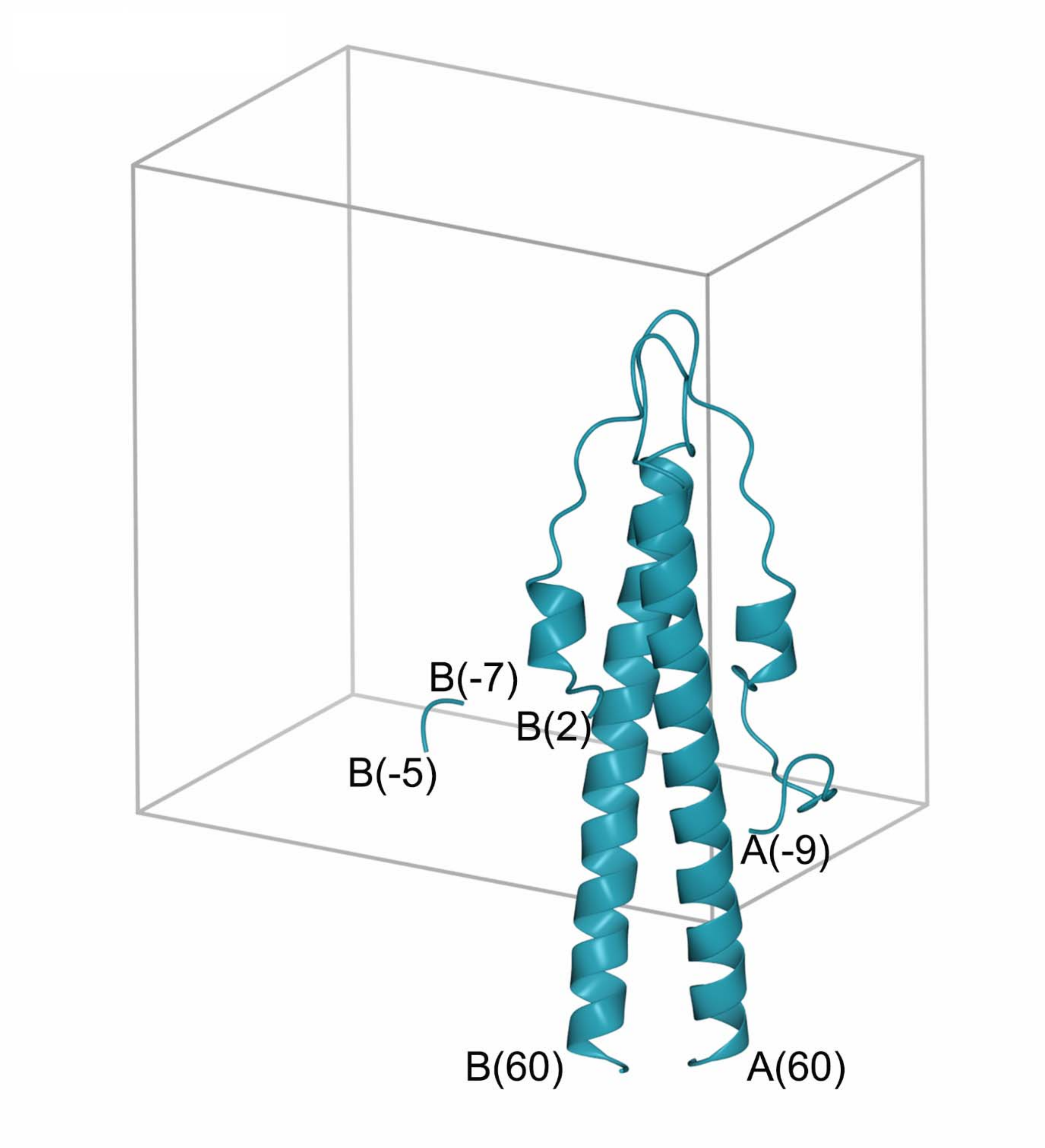

Figure S1 Structure of N-Wag31. The monoclinic unit cell is shown as a box. N-Wag31 dimer within the asymmetric unit, including the poly-histidine tag regions (see experimental section), is shown as a cartoon in blue.

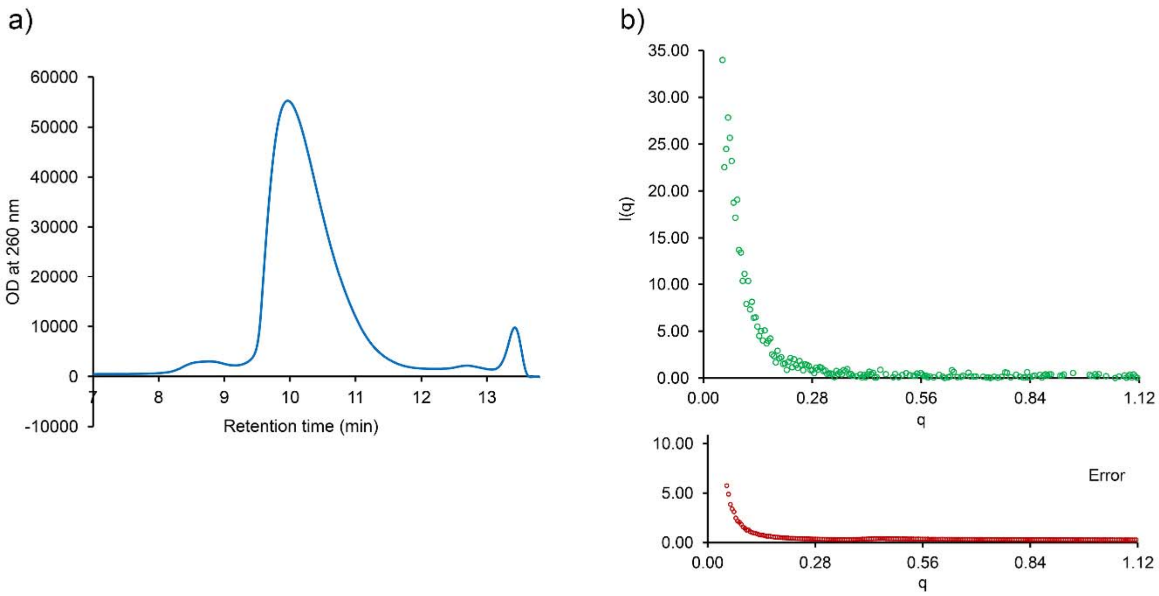

Figure S2 SEC-SAXS data of N-Wag31. (a) UV elution profile (UV absorbance in arbitrary unit at 260 nm wave-length *versus* retention time in minutes) of N-Wag31 during the SEC-SAXS experiment. (b) Intensity (I) *versus* momentum transfer (q in nm^{-1}) for averaged SAXS intensity profile, and associated error, obtained from the peak I region shown in figure 3a.
